# Grooming Future Physician-scientists: Evaluating the Impact of Research Motivations, Practices, and Perceived Barriers Towards the Uptake of an Academic Career Among Medical Students

**DOI:** 10.7759/cureus.1991

**Published:** 2017-12-27

**Authors:** Sayed Mustafa Mahmood Shah, Mahnoor Sohail, Khwaja Mubeen Ahmad, Fouzia Imtiaz, Sundus Iftikhar

**Affiliations:** 1 Department of Internal Medicine, Dow University of Health Sciences (DUHS), Karachi, Pakistan; 2 Biochemistry, Dow University of Health Sciences (DUHS), Karachi, Pakistan; 3 The Indus Hospital Research Center, The Indus Hospital

**Keywords:** medical student research, career choice, motivation, barriers, interventions, medical curriculum, undergraduate medical

## Abstract

Purpose: To evaluate the research trends and underlying motivations that shape intentions for the future uptake of an academic career among medical students. Further, to investigate the barriers and sought-after interventions which may optimise research outcomes in a resource-limited setting.

Methods: A cross-sectional study was conducted among 294 undergraduate (UG) medical students in Karachi, Pakistan. A self-administered questionnaire was employed to assess current research practices and future intentions, and to evaluate related motivations, barriers, and sought-after interventions.

Results: Almost two-thirds of medical students reported some form of involvement in medical research and expressed positive attitudes towards the same. However, intentions to pursue research at a professional level not only remained low (19.7%) but were found to decrease with each passing year of study (p<0.01). The most commonly expressed motivation for pursuing research was “admission into a residency program” (71.8%), and was associated with a decreased likelihood of pursuing research professionally. The most cited barriers to conducting UG research were a “lack of time” (72.4%), “lack of supervisors” (50.3%) and a “lack of opportunities in the university” (48.3%). A dichotomy in sought-after interventions was observed among research-naïve and research-experienced students.

Conclusions: Despite promising trends in UG medical research, the intentions for uptake of an academic career remain low. Research practices driven by career enhancement alone may be detrimental. Interventions to increase research output must promote the capacity building of research-naïve students and facilitate the ongoing practices of research-experienced students.

## Introduction

Research has been the fundamental cornerstone upon which science progresses and new findings are unearthed for knowledge. Knowledge has to be generated at a university through research and inquiry [1]. Accordingly, medical research becomes even more crucial to gain greater knowledge of all existing and adapting disease processes and to develop improved methods to prevent and fight illness through the betterment of modern medical practices. The role of the physician-scientist is vital. Conducting medical research not only develops clinical methods but also makes physicians capable of questioning, interpreting, and analyzing situations.

The provision of adequate medical services is hinged upon the association of physician-scientists and clinicians. A delicate balance between the works of these two professional practices is essential for the advancement of current medical knowledge, upon which the framework of clinical practice is further given shape. A dearth of individuals contributing to the field of medical research would bring the evolution of clinical practice to a standstill. Innovations in medical diagnosis and treatment are due to the advancement in different fields of science by successful research projects [2].

Furthermore, research at the undergraduate (UG) level inculcates crucial skills and habits such as the ability to question, explore, investigate, interpret, and analyse. These characteristics are essential in developing the student’s reliance on detailed thought process which is the basis of making a reliable clinician. Scholarly activity programs are essential components of the modern UG medical curriculum [3].

Despite promising trends in research practices, attitudes, and uptake of knowledge among medical students in varied settings, the academic impact of student-driven research activities remains questionable [4-7]. Moreover, a shortfall of physician-scientists persists, particularly in South Asia where there is a distinct lack of research infrastructure and policies for UG medical students [8-9].

This study was conducted to assess ongoing research trends among medical students in a teaching hospital in Karachi, Pakistan and to investigate factors influencing intentions to pursue an academic career among UG medical students. Moreover, barriers towards conducting UG medical research and sought-after interventions were also evaluated to guide targeted interventions in a setting where research infrastructure is limited.

## Materials and methods

A descriptive cross-sectional study was conducted at the Dow Medical College, Karachi to investigate the attitudes, knowledge, and practices of UG medical students towards research as well as their perceived motivations, barriers, and sought-after interventions for the UG medical research in Pakistan. The study period spanned five months from 1st August, 2016 till 1st December, 2016. The target population comprised of all UG medical students; i.e., those enrolled in a five-year Bachelor of Medicine, Bachelor of Surgery (MBBS) program, studying at the Dow Medical College, from the first year to the fifth year. All students who had completed the UG training, those who were enrolled in courses other than MBBS, and those not enrolled in at the Dow Medical College were excluded.

Open Epi was employed to determine the required sample population for this study under a 95% confidence interval, which was found to be 384. However, we opted for a sample of 400. The target population was approached using a purposive/judgmental sampling of 80 individuals per each year of study, thus comprising a total sample population of 400 students from the first year to the fifth year [10].

The questionnaire used in this study was devised following an extensive literature review on the subject matter, and under the guidance of experienced faculty members in the field of Community Medicine and Medical Research. The questionnaire assessed the attitudes, knowledge, and practices of UG medical students towards research. Moreover, it evaluated their perceived motivations, barriers, and sought-after interventions for conducting medical research. Lastly, the future intentions for conducting UG medical research and following a career in medical research were inquired. Participant responses to questions regarding attitudes, knowledge, and future intentions pertaining to UG medical research were recorded using a three-point Likert scale. Responses to perceived motivations, barriers, and sought-after interventions were recorded using a structured checklist; it was represented graphically as a percentage of the total participant responses (n=294).

To ensure the validity of our questionnaire and hence reduce bias, we conducted a pilot study on a convenience sample of 20 students (5% of the total sample). Following the pilot study, any difficult to answer or understand questions were amended for clarity. All forms from the pilot study were excluded from the final analysis.

Data collection was initiated with the validated and pre-tested 18 item questionnaire. Prior to administering the questionnaires, a brief description of the study objectives was provided and informed; written consent was acquired from all willing participants. Confidentiality and anonymity of all study participants were maintained throughout the study.

Our response rate was 92%. However, following removal of incomplete or incorrectly filled forms, we were left with a total of 294 questionnaires for analysis. As such, no imputation models were employed.

Data was analysed using Statistical Package for Social Sciences (SPSS version 20.0) (IBM Corp., Armonk, NY). Categorical variables were expressed using frequencies and percentages. Continuous variables were presented as mean and standard deviation. Pearson chi-squared test and multiple-response chi-squared test with a 95% confidence interval were used to compare categorical variables.

## Results

Of the 294 individuals who participated in this study, approximately three-quarters were females (n=225) and one quarter were males (n=69) (Table 1). The distribution of study participants across the year of study (YOS) is shown in Table 1.

**Table 1:** Demographic characteristics of study participants

Demographic characteristics		Number (%)
Gender	Males	69 (23.5)
	Females	225 (76.5)
Year of study	1^st^ Year	54 (18.4)
	2^nd^ Year	70 (23.8)
	3^rd^ Year	56 (19.0)
	4^th ^Year	64 (21.8)
	5^th^ Year	50 (17.0)

While a clear majority of the study participants acknowledged the positive role medical research plays in their future as clinicians (90.1%), less than half (48.8%) felt that conducting medical research would pose no hindrance to their UG studies.

When asked to rate their current level of knowledge regarding research methodology, most participants claimed to have “no” (8.9%) or “slight knowledge” (46.8%) in this regard. Only a minority claimed to have “excellent knowledge” of the above (4.8%), whereas the rest claimed to have “moderate knowledge” (39.6%). It was found that males expressed superior knowledge as compared to females (p=0.005). Moreover, knowledge was shown to be greater in participants with a higher YOS (p=0.000).

The research practices of study participants are shown in Table 2. Males and females had approximately equal levels of involvement in UG medical research (p=0.491), with a greater tendency for pursuing voluntary research among male participants (p=0.044). Males were also significantly more likely to have published an article than females (p=0.001) (Table 2).

**Table 2:** Research practices of study participants; stratified by gender and year of study

	Involved in medical research Number (%)	Nature of research involvement			Journal publications Number (%)	Presented at seminars Number (%)
		Voluntary Number (%)	Compulsory Number (%)	Both types Number (%)		
Overall	185 (62.9)	87 (47.0)	68 (36.8)	30 (16.2)	25 (13.5)	20 (10.8)
Males	41 (59.4)	26 (63.4)	9 (22.0)	6 (14.6)	13 (31.7)	7 (17.1)
Females	144 (64.0)	61 (42.4)	59 (41.0)	24 (16.7)	12 (8.3)	13 (9.0)
1^st^ Year	7 (13.0)	6 (85.7)	0 (0.0)	1 (14.3)	3 (42.9)	4 (57.1)
2^nd^Year	34 (48.6)	32 (94.1)	2 (5.9)	0 (0.0)	2 (5.9)	1 (2.9)
3^rd ^Year	32 (57.1)	30 (93.8)	2 (6.2)	0 (0.0)	2 (6.3)	1 (3.1)
4^th^ Year	63 (98.4)	12 (19.0)	33 (52.4)	18 (28.6)	3 (4.8)	3 (4.8)
5^th^ Year	49 (98.0)	7 (14.3)	31 (63.3)	11 (22.4)	15 (30.6)	11 (22.4)

Research involvement increased dramatically with YOS, with greater than 98% of fourth and fifth-year students expressing research involvement at the time of the study (p=0.000). Students from first to third years participated predominantly in voluntary research, whereas compulsory research comprised the bulk of research practices among the fourth and fifth years (p=0.000) (Table 2). Final year students were found to have had the most research publications (n=15).

While a significant proportion of students expressed a desire to pursue UG medical research (67%), less than one fifth was intent on pursuing medical research as a career path (19.7%), as shown in Table 3. Males were more likely than females to express future intent to pursue medical research professionally (p=0.022). Intent to pursue medical research, both at a UG level and professionally, was seen to decrease with higher YOS (p=0.000) (Table 3).

**Table 3:** Future research intentions of study participants at the undergraduate and professional levels; stratified by gender and year of study

	Intent on pursuing medical research at the undergraduate level Number (%)		Intent on pursuing medical research as a career path Number (%)	
Overall	197 (67.0)		58 (19.7)	
Males	48 (69.6)	p=0.847	21 (30.4)	p=0.022
Females	149 (66.2)		37 (16.4)	
1^st^ Year	35 (64.8)	p=0.000	16 (29.6)	p=0.000
2^nd^Year	57 (81.2)		16 (22.9)	
3^rd^ Year	41 (73.2)		9 (16.1)	
4^th^ Year	38 (59.4)		11 (17.2)	
5^th ^Year	26 (52.0)		6 (12.0)	

Significant associations were found between intentions to pursue medical research at the UG Level and positive attitudes towards research (p<0.005), knowledge regarding research (p=0.010), prior voluntary research practices (p=0.000) and having read medical literature (p=0.000). Predictors of future intent to pursue medical research as a career path included: a belief that medical research would not pose a hindrance to studies (p=0.001), prior research involvement (p=0.003), and a desire to pursue medical research at the UG Level (p=0.000).

**Figure 1:**
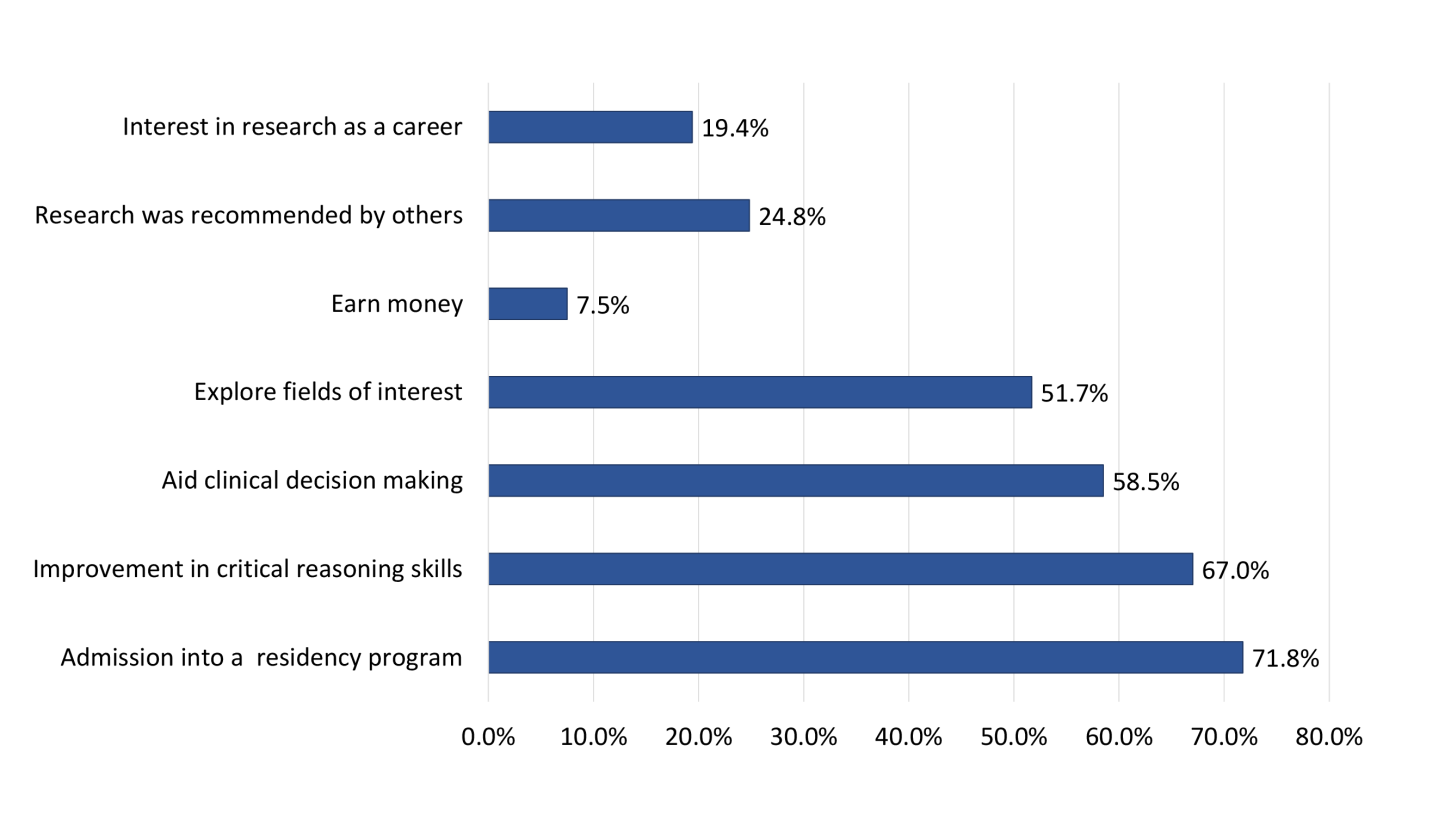
Perceived motivations for conducting undergraduate medical research among study participants

The most cited motivations for pursuing UG medical research included “admission into a residency program” (71.8%), “improvement in critical thinking skills” (67.0%), and to “aid clinical decision making” (58.5%), as shown in Figure 1. It was observed that those who did not report a future intention to pursue medical research as a career path were more likely to report “admission into a residency program” (78.3%) as a motivation, compared to those who reported a future intention to pursue medical research professionally (70.7%) (p=0.000).

**Figure 2:**
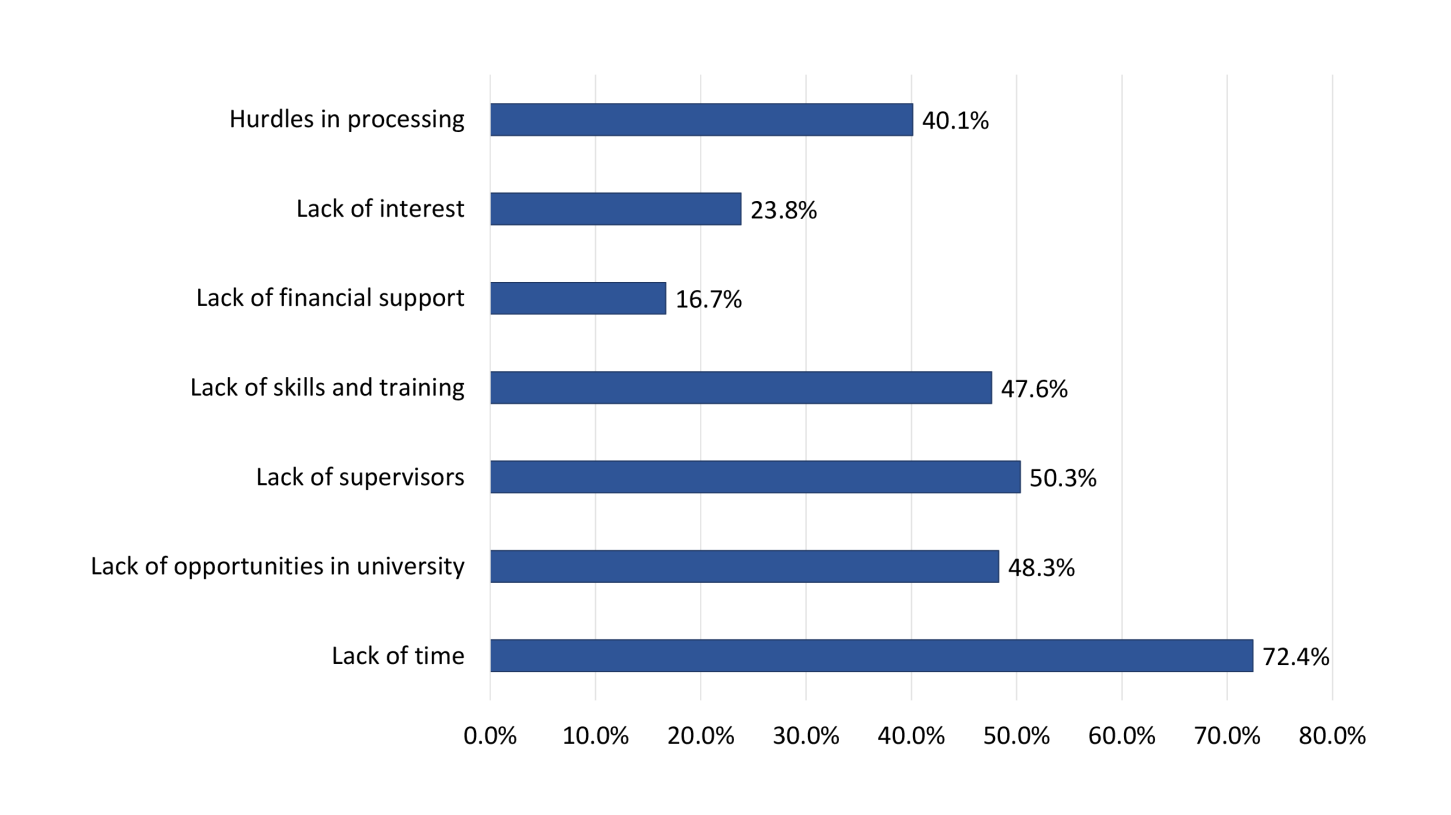
Perceived barriers towards conducting undergraduate medical research among study participants

The most cited barriers to conducting UG medical research were: “lack of time” (72.4%), “lack of supervisors” (50.3%), and a “lack of opportunities in the university” (48.3%) (Figure 2). When stratified by research involvement, students already involved in research were most likely to cite “lack of time” (72.4), “lack of supervisors” (54.1%) and “hurdles in processing” (46.5%) as barriers, whereas those with no prior research experience cited “lack of time” (72.5%), “lack of opportunities in the university” (56.0%) and “lack of skills and training” (53.2%) as barriers (p=0.010).

**Figure 3:**
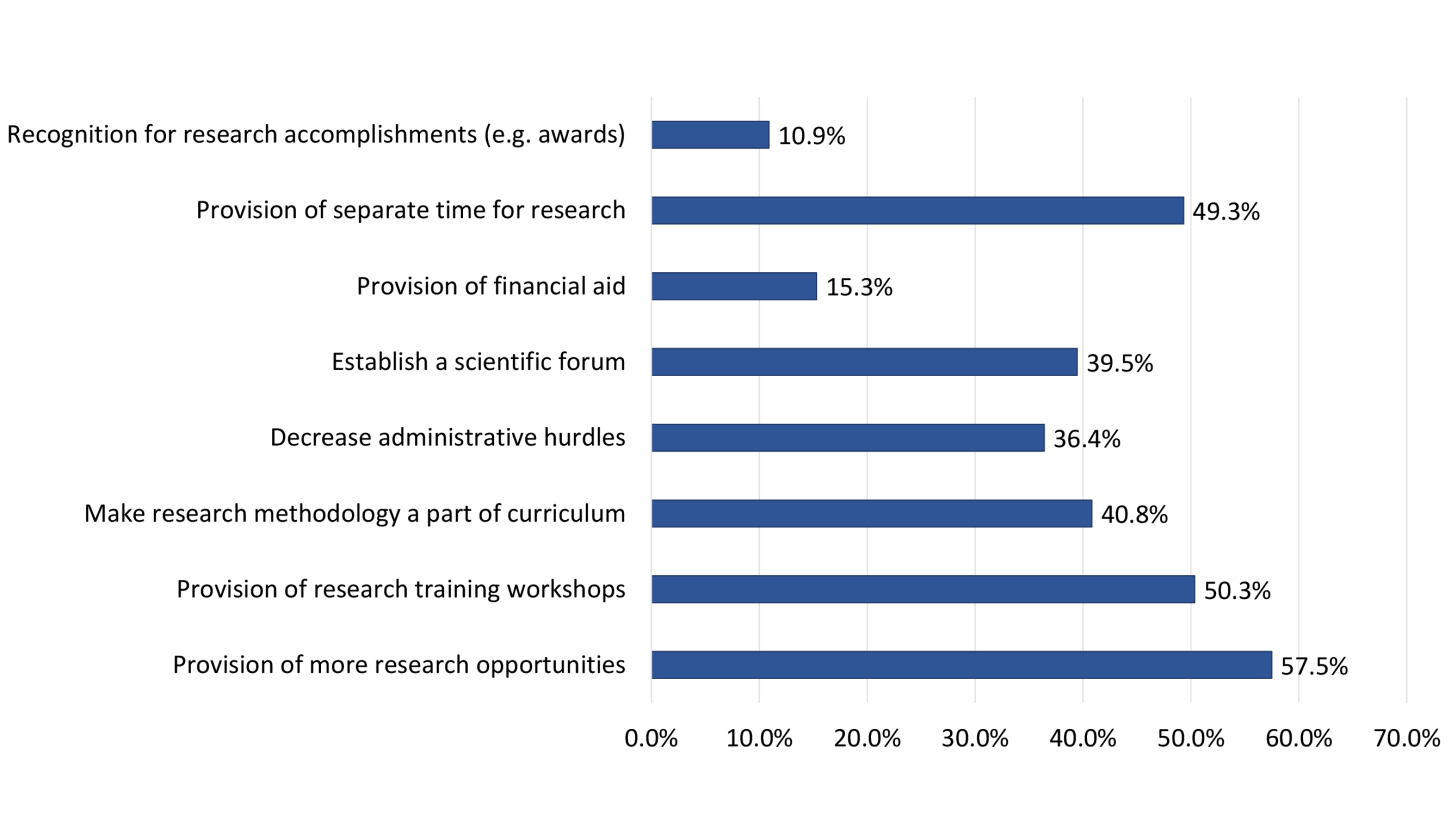
Most sought after interventions for promoting undergraduate medical research among study participants

The most sought-after interventions to promote UG medical research included: “provision of more opportunities” (57.5%), “provision of research training workshops” (50.3%) and “provision of separate time for research” (49.3%) (Figure 3). It was found that those with prior research experience were more likely to seek interventions such as “decrease administrative hurdles” (41.1%), “establish a scientific forum” (41.6%) and “provision of financial aid” (18.4%). In contrast, those without prior research experience were more likely to seek research training in the form of “research training workshops” (56.9%) or research methodology in the curriculum (47.7%) (p=0.021).

## Discussion

In this cross-sectional study, we observed that despite promising trends in UG research knowledge, and practices, future intentions to pursue medical research professionally not only remained low but were found to decrease over time. Medical research in South Asian countries is an under-developed and neglected field, which has far-reaching ramifications for the populations residing therein [9]. The engagement and grooming of medical students to pursue medical research has been advocated as an effective strategy to counterbalance the growing decline in physician-scientists and help developing countries reach self-reliance in health research [4, 9].

Despite encouraging reports of medical students’ growing interest in research, these trends have shown to be a weak indicator of future research productivity [4-7, 11]. The uptake of an academic career among medical students is undeniably a complex and dynamic process, shaped by the cumulative effect of past experiences and related behavioural domains [6]. To systematically address the various factors influencing research uptake among medical students, a theoretically informed approach has been proposed [12]. According to said approach, the factors influencing uptake of research among medical students may be grouped into three behavioural domains: autonomy, competence, and relatedness [12]. Our results support the applicability of a theoretical framework in this setting and further guide the adoption of interventions aimed at improving research output.

We observed that future intentions to pursue medical research at a UG level was associated with positive attitudes towards research, knowledge regarding research, and prior voluntary research experiences (p<0.005). While at the first glance, the significant proportion of students expressing intentions to pursue UG medical research appears re-assuring (67.0%), further scrutiny shows the intentions for the same were found to decrease with each passing year of study (p<0.05). Similarly, final year students, in whom superior research practices and knowledge were demonstrated, reported the least intent to pursue medical research at a UG level (52.0%) and professionally (12.0%). These conflicting results may be a product of misdirected motivations. The strongest reported motivation for conducting UG medical research in our cohort was “to facilitate admission into a residency program” by 71.8% of respondents. However, our analysis showed that such motivations were associated with a decreased intent to pursue medical research professionally. These findings correspond to the observations of Rosenkranz, et al. on autonomy, whereby extrinsic motivators, such as career advantage alone, can demoralise and damage future prospects of research uptake among medical students [12].

The most significant barriers to conducting UG research by our study respondents included “lack of time” (72.4%), “lack of supervisors” (50.3%) and “lack of opportunities” (48.3%). Similar findings have been reported by multiple studies among medical students and physicians alike [6, 9, 13-16]. A novel finding in our setting was the dichotomy in reported barriers and sought-after interventions by research-naïve and research-experienced students. It was observed that students without prior research involvement were more inclined to seek interventions which focused on capacity building and developing the related competence of the individual, via “provision of training workshops” and “provision of research methodology in the curriculum”. In contrast, those with prior research experience were more likely to seek interventions aimed at facilitating pre-existing research engagements, such as “decreasing administrative hurdles”, “provision of financial aid” and facilitated mentor access via “establishment of a scientific forum”. Similar dichotomies in perceived barriers to conducting medical research have been reported among junior faculty members from teaching hospitals in Pakistan, and illustrate the need for tailoring future interventions to the needs and expectations of the target population [4].

At our institution, involvement in compulsory research projects becomes a curricular requirement for students in clinical years of study; third year to the fifth year. We observed that a greater intent to pursue medical research at UG and professional levels was associated with voluntary research involvement, rather than compulsory curricular requirements. Amgad, et al. reported similar findings in his systematic review and meta-analysis whereby several reports emphasised superior research outcomes in voluntary research, as opposed to curriculum mandated research [17]. Nevertheless, compulsory research activities have been advocated as an effective and accessible means for capacity building of research-naïve students, facilitating their recruitment into research projects and developing an awareness for evidence-based medicine [12, 18]. The success of such programs undoubtedly depends on their execution in a time protected and structured manner, alongside adequate mentorship [19-21]. This is reflected by the top three reported barriers to conducting medical research by our study respondents as: “lack of time”, “lack of supervisors” and “lack of research opportunities”.

A notable finding was the apparent gender disparity with respect to research knowledge, publications, and future career intentions, with males demonstrating superior outcomes over females. While Amjad, et al. in his review also reported a greater likelihood of publishing among male medical students, no gender disparity was noted with respect to research knowledge/ skills, research attitudes, or interest in research as a career [17]. These results must be interpreted under the prevailing socio-cultural contexts. Gender-specific barriers to conducting UG medical research have been prominently reported in conservative settings such as Saudi Arabia where female students are hindered by a lack of access to patients/samples and a paucity of same-sex mentors/role models [13-14]. Future interventions in conservative and Eastern settings, such as ours, should therefore, offer an additional component of gender-specific training to enable female students to perform research in a culturally sensitive and effective manner.

Despite our best efforts, this study is subject to limitations imposed by the study design. The first of these is the non-randomised selection of study participants from a single centre, which limits generalisability. Secondly, due to the cross-sectional nature of this study, we cannot derive any causal relationships and our findings remain a hypothesis. Lastly, we assessed key outcomes such as research knowledge and future intentions by self-report which may be subject to over or underestimation [14].

## Conclusions

Despite promising trends in medical students’ knowledge and involvement in UG research, future intentions to pursue research professionally remain low. Research involvement driven by career advancement alone may be detrimental to the future uptake of research careers. Effective uptake of research among medical students must be guided by a theoretically informed approach comprising of capacity building for research-naïve students and facilitation of those with pre-existing research commitments. Gender-specific training for females and promotion of female role models in this setting is recommended.
